# Dr. Everett W. Fish

**Published:** 1912-04

**Authors:** 


Dr. Everett W. Fish of Pittsford, died suddenly, March 24, 1912, aged 66. He was a graduate of the University of Michigan but had not practiced medicine for many years, having engaged mainly in newspaper work.

